# Methylation of the CpG Sites Only on the Sense Strand of the *APC* Gene Is Specific for Hepatocellular Carcinoma

**DOI:** 10.1371/journal.pone.0026799

**Published:** 2011-11-02

**Authors:** Surbhi Jain, Ting-Tsung Chang, James P. Hamilton, Selena Y. Lin, Yih-Jyh Lin, Alison A. Evans, Florin M. Selaru, Pin- Wen Lin, Shun-Hua Chen, Timothy M. Block, Chi-Tan Hu, Wei Song, Stephen J. Meltzer, Ying-Hsiu Su

**Affiliations:** 1 Department of Microbiology and Immunology, Drexel University College of Medicine, Philadelphia, Pennsylvania, United States of America; 2 Department of Medicine, Infectious Diseases and Signaling Research Center, College of Medicine, National Cheng Kung University, Tainan, Taiwan, Republic of China; 3 Division of Gastroenterology and Hepatology, Department of Medicine, The Johns Hopkins University School of Medicine, Baltimore, Maryland, United States of America; 4 Department of Oncology, The Sidney Kimmel Comprehensive Cancer Center, Baltimore, Maryland, United States of America; 5 Department of Surgery, National Cheng Kung University Hospital, Tainan, Taiwan, Republic of China; 6 School of Public Health, Drexel University, Philadelphia, Pennsylvania, United States of America; 7 Department of Microbiology, Medical College, National Cheng Kung University, Tainan, Taiwan, Republic of China; 8 Department of General Medicine, Buddhist Tzu Chi General Hospital, Hualien, Taiwan, Republic of China; 9 JBS Science Inc., Philadelphia, Pennsylvania, United States of America; John Hopkins Medical School, United States of America

## Abstract

Hypermethylation of the promoter of the tumor suppressor gene, adenomatous polyposis coli (*APC*), occurs in various malignancies, including hepatocellular carcinoma (HCC). However, reports on the specificity of the methylation of the *APC* gene for HCC have varied. To gain insight into how these variations occur, bisulfite PCR sequencing was performed to analyze the methylation status of both sense and antisense strands of the *APC* gene in samples of HCC tissue, matched adjacent non-HCC liver tissue, hepatitis, cirrhosis, and normal liver tissues. DNA derived from fetal liver and 12 nonhepatic normal tissue was also examined. These experiments revealed liver-specific, antisense strand-biased CpG methylation of the *APC* gene and suggested that, although methylation of the antisense strand of the *APC* gene exists in normal liver and other non-HCC disease liver tissue, methylation of the sense strand of the *APC* gene occurs predominantly in HCC. To determine the effect of the DNA strand on the specificity of the methylated *APC* gene as a biomarker for HCC detection, quantitative methylation-specific PCR assays for sense and antisense strand DNA were developed and performed on DNA isolated from HCC (n = 58), matched adjacent non-HCC (n = 58), cirrhosis (n = 41), and hepatitis (n = 39). Receiver operating characteristic curves were constructed. With the cutoff value set at the limit of detection, the specificity of sense and antisense strand methylation was 84% and 43%, respectively, and sensitivity was 67.2% and 72.4%, respectively. This result demonstrated that the identity of the methylated DNA strand impacted the specificity of *APC* for HCC detection. Interestingly, methylation of the sense strand of *APC* occurred in 40% of HCCs from patients with serum AFP levels less than 20 ng/mL, suggesting a potential role for *APC* as a biomarker to complement AFP in HCC screening.

## Introduction

Hepatocellular carcinoma (HCC) is the fourth leading cause of cancer and the third leading cause of cancer deaths in the world. Although the 5-year survival rate for patients with liver cancer is 14%, it increases to 26% in patients in whom cancer is found at an early stage, compared with only 2% when it is found after it spreads to distant organs [1]. Unfortunately, it is difficult to detect HCC early using current screening methods.

Cancer is a disease of the genome; thus, detection of genetic or epigenetic changes underlying HCC development should provide unambiguous tumor detection at a curative stage [2]. Methylation of multiple tumor suppressor genes has been shown to play a role in the genesis of HCC [3], [4], [5], [6]. These hypermethylation markers offer promise as tools to detect cancer cells in tissue and body fluids [7], [8] with the use of simple PCR technology [9], [10], [11]. Proof of principle for the clinical value of methylation markers has been reported for the early detection and classification of cancer [12], [13], [14], [15], [16], [17], [18], [19], [20], [21], risk assessment and prognosis [19], [22], [23], [24], and prediction of therapy response [25], [26], [27], with some already having shown their importance in (pre)clinical practice. Thus, the use of methylation markers as a powerful diagnostic and predictive tool is becoming a reality [7].

Recent evidence, as reviewed by van Vlodrop, has implicated the location of aberrant CpG dinucleotide methylation on gene expression and on its clinical value in cancer [28]. This work suggests that the current data on hypermethylation markers require a more comprehensive and critical evaluation prior to its implementation in clinical practice.

Inactivation of one of the many tumor suppressor genes, the adenomatous polyposis coli (*APC*) gene, by genetic or epigenetic modifications, particularly methylation, appears to be a key event in several cancers, including HCC [4], [5], [26], [29], [30], [31], [32]. Although an association between HCC and the hypermethylation of the *APC* gene (*mAPC*) has been described in more than 30 publications, the degree of association varies among these studies, as shown in Table 1. As indicated, some studies reported up to 90% association of *mAPC* with HCC and no *mAPC* in non-HCC livers [4], [6], [32], whereas others report a predominance of *mAPC* in both HCC and non-HCC liver samples [33], [34], [35]. Interestingly, the primary variable in these studies was the DNA strand analyzed. That is, either the sense strand or the antisense strand was examined (Table 1). We thus hypothesized that the variable specificity of *mAPC* for HCC is due to differences in the methylation status between the sense and antisense strands, suggesting the existence of strand-specific bias in the methylation of the promoter and the first exon regions of the *APC* gene in liver tissue.

**Table 1:** Comparison of primer locations and association between APC promoter methylation and HCC in previously published articles.

Study[reference]	Primer location	Assayformat	Percent of tissue methylated (sample size)	
			Non-HCC	HCC
1 [4]	Sense	MSP	0 (20)	81.7 (60)
2 [32]	Sense	MSP	14.3 (14)	53 (51)
3 [6]	Sense	MSP	NA (95% specificity, n = 20 for HCC and non-HCC tissue)	
4 [34]	Antisense	MethyLight-Taqman	81 (19)	100 (19)
5 [35]	Antisense	MethyLight-Taqman	48 (25)	78 (40)
6 [33]	Antisense	Quantitative MSP	100 (16)	100 (34)

Note: NA, not applicable.

In this study, we performed comprehensive bisulfite-specific PCR (BSP) sequencing of the sense and antisense strands of 250 base pairs (bp) of the promoter and the first exon region of the *APC* gene followed by quantitative methylation-specific PCR assays of both DNA strands on DNA isolated from HCC, matched adjacent non-HCC, cirrhosis, hepatitis, and normal liver tissues. We demonstrated that the extent of HCC specificity of *mAPC* varies with the DNA strand analyzed. Although the existence of liver-specific antisense strand-biased methylation in non-HCC includes normal liver tissue, methylation of both sense and antisense strands is evident in HCC tissue. This finding indicates that only sense-strand methylation is specific to HCC when an end-point methylation-specific PCR (MSP) assay is used for analysis.

## Results

### Antisense strand-biased methylation of the promoter and first exon regions of the *APC* gene in human liver tissue determined by bisulfite-specific PCR sequencing

**Figure 1:**
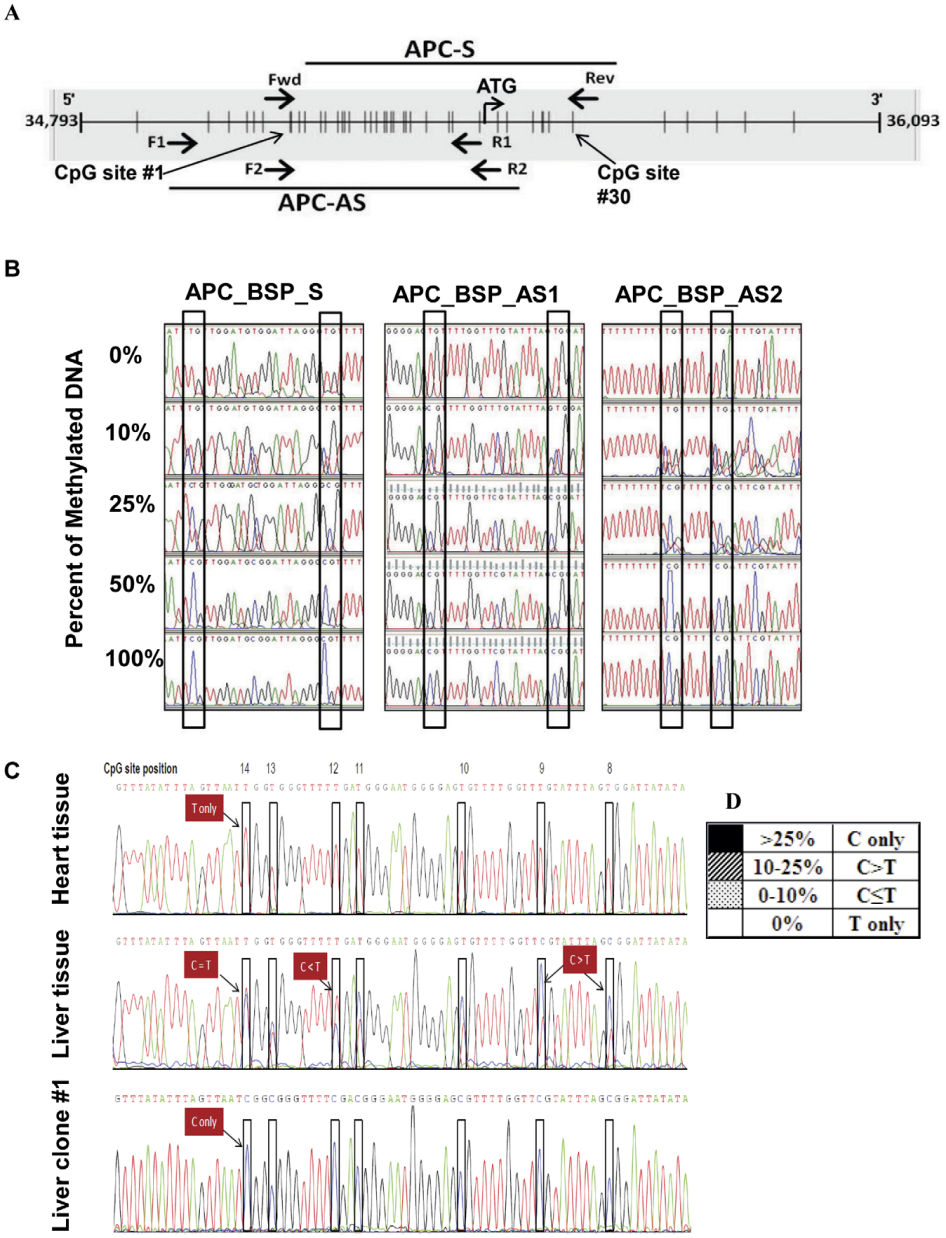
Establishment of a reference index for BSP sequencing. A, diagram of the locations of bisulfite sequencing primers and the CpG sites, indicated by vertical bars, in the promoter and the first exon regions of the *APC* gene (Genebank accession # NG_0084811, nt. 34,793—36,093). The ATG site is also indicated. The CpG sites bracketed by the bisulfite sequencing primers for the sense strand *“APC-S”* and the antisense strand “*APC*-AS” were numbered from 1 to 30 on the basis of the sense strand 5′ to 3′ direction. B, representative chromatograms of BSP sequencing of the reconstituted standards: 0% methylated+100% unmethylated DNA (0%);10% methylated DNA+90% unmethylated DNA (10%); 25% methylated DNA+75% unmethylated DNA (25%); 50% methylated DNA+50% unmethylated DNA (50%); and 100% methylated DNA (100%), from three pairs of bisulfite specific primers, APC_BSP_S, APC_BSP_AS-1, and APC_BSP_AS-2, as indicated. The boxed areas show the relative “C” and “T” peaks in the chromatogram from each sample of the reconstituted standards by each primer set as indicated. C, the index for analysis of the methylation status of each CpG site based on the DNA sequencing chromatograms obtained from heart, liver, and one liver bisulfite-specific PCR clone, ranging from CpG site #8 to #14. At each CpG site, indicating four possible “C/T” peaks and summarized in a reference index (D) based on the analysis from panel B, as following: (1) only C was detected (C only, black box, indicating methylation); (2) the C peak was higher than the T peak (C>T, hatched box); (3) the C peak was equal to or lower than the T peak (C = T & C<T, dotted box); and (4) only T was detected (T only, open box, indicating no methylation).

To test the hypothesis that the variable HCC specificity of *mAPC* found in previous studies was due to differences in methylation status between the sense and antisense strands of the *APC gene* in normal and diseased liver tissues, we designed BSP primers to determine the methylation status of the sense and antisense strands of *APC* by BSP sequencing. Figure 1A shows CpG sites (vertical bars) in the promoter and first exon regions of the *APC* gene, along with locations of BSP primers (primer sequences are listed in Table S1). Thirty CpG sites within the 575-bp region studied were numbered from 1 to 30 in the 5′ to 3′ direction.

To control for the efficiency of the bisulfite conversion, we determined the percentage of cytosine (C) to thymine (T) conversions that occurred in non-CpG cytosines within the analyzed region after DNA sequencing of BSP product from each sample. Only samples yielding a C-to-T conversion rate higher than 95% for these non-CpG Cs were analyzed further.

To analyze the extent of methylation at each CpG site by BSP sequencing, we established a reference index (Fig. 1C and 1D) on the basis of the BSP sequencing data of the reconstituted standards (Fig. 1B). We categorized the ^m^CpG into four groups: (1) T only (0% methylation detected, open boxes); (2) C less than or equal to T (^m^C detected on less than 10% of total DNA, dotted boxes); (3) C greater than T (^m^C detected on ∼10%–25%, hatched boxes); (4) C only (^m^C detected on ∼25% or more of the DNA, solid boxes), as shown in Figure 1D and illustrated in the chromatograms from the BSP sequencing of normal liver and heart and a BSP clone derived from normal liver DNA (Fig. 1C).

**Figure 2:**
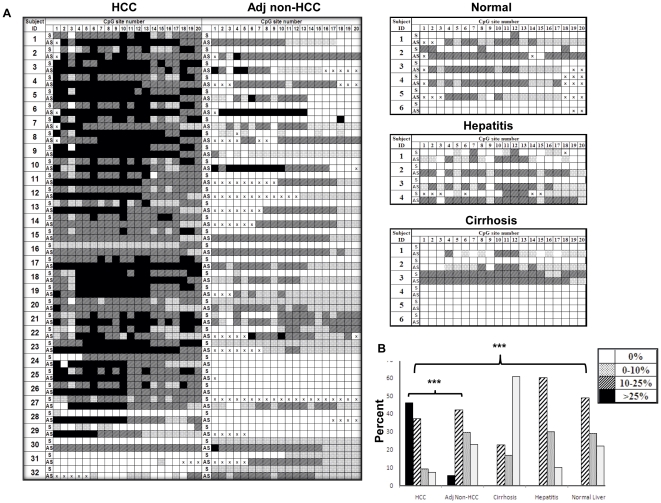
Methylation profiles of the sense and antisense strands of the *APC* gene by BSP sequencing of DNA isolated from normal liver and diseased liver tissues. A, methylation status of each CpG site in both sense (S) and antisense (AS) strands of the promoter and the first exon regions of the *APC* gene in hepatocellular carcinoma (HCC, n = 32) tissue, matched adjacent non-HCC liver tissue (Adj Non-HCC, n = 32), and normal (n = 6), hepatitis (n = 4), and cirrhosis (n = 6) tissues. The data were analyzed as described in Figure 1. Because of the large amount of T in the DNA template after bisulfite conversion, sequencing results from some CpG sites were not available and are designated as “x.” B, histogram comparing the density of ^m^CpG detected in the antisense (AS) strand of the *APC* gene in HCC vs. adjacent non-HCC (***P<0.0001), and HCC vs. normal liver (***P<0.0001) tissue by BSP sequencing analyzed as shown in panel A.

BSP sequencing was performed on bisulfite-treated DNA samples from HCC (n = 32), matched adjacent non-HCC (n = 32), normal liver (n = 6), hepatitis (n = 4), and cirrhotic tissues (n = 6) as described in Materials and Methods. The demographic profiles of the subjects are summarized in Table 2. The BSP sequencing data were analyzed using the reference index established by the reconstituted standards (Fig. 1). Because the presence of one 16–bp poly-T sequence in the antisense strand after bisulfite conversion rendered it unreadable by sequencing after CpG site 20, we only analyzed CpG sites 1 to 20 (Fig. 2). As expected, we detected ^m^CpG in both the sense and antisense strands of the *APC* gene in most HCC DNA samples (27/32). Interestingly, we observed an antisense-biased methylation pattern in matched adjacent non-HCC tissue (19/32), in normal liver (5/6) samples, and in samples of liver from patients with hepatitis (3/4) and cirrhosis (2/6). The methylation of the sense strand for these non-HCC tissues was not detected by BSP sequencing whereas the antisense strand showed variable densities of methylation. One cirrhosis sample, C3, showed symmetrical methylation, whereas two others, C4 and C5, showed no methylation (Fig. 2A).

**Table 2:** Summary of clinicopathological characteristics of the tissues analyzed by BSP.

Characteristic	Normal*N = 6	Hepatitisn = 4	Cirrhosisn = 6	HCC and adjacent non-HCCn = 32
**Mean age ± SD** ≠ **, years**	64±7.5	60±13.39	51±18.53	58±11.11
**Male/female**	4/2	2/2	4/2	20/12
**HBV/HCV/HBV and HCV/nonviral or unknown**	0/0/0	0/0/0	0/5/1	20/7/1/4
**Stage** § **1/2/3/4/unknown**	-	-	-	14/7/0/1/10
**Grade 1/2/3/unknown**	-	-	-	6/13/3/10
**Mean size of tumor ± SD** ≠	-	-	-	5.6±3.6
**AFP levels (ng/ml)** **≤20/>20/unknown**	-	-	-	12/10/10

*4 of these 6 normal livers are normal liver tissues with concomitant cholangiocarcinoma.

§ HCC tumors were staged using the tumor-node-metastasis (TNM) staging system.

≠ SD, standard deviation.

To test our hypothesis, we determined the specificity of the *mAPC* sense strand and antisense strand to HCC based on the BSP sequencing data (Fig. 2A). We defined the “positive” as the DNA strand in which methylation was detected in ≥50% of CpG sites analyzed by BSP sequencing. The specificity of *mAPC* sense and antisense strands to distinguish HCC from all non-HCC tissue, including normal liver, hepatitis, cirrhosis, and adjacent non-HCC, was 79% and 19%, respectively, suggesting that methylation of only the sense strand of the *APC* had significant HCC specificity.

### Methylation density of the antisense strand in HCC tissues was significantly higher than that in non-HCC tissues

Next we analyzed the methylation density at the level of the CpG site of the antisense strand of the *APC* promoter and first exon regions by BSP direct sequencing. The number of CpG sites of each of four categories was calculated. The overall percentage of ^m^CpG detected in the antisense strand of the *APC* gene among each disease category of liver tissue samples is summarized in Table S2 and plotted in Figure 2B. We did not detect any “C only” (filled boxes) in a total of 295 CpG sites analyzed (0/295) in normal, hepatitis, or cirrhosis liver samples, whereas 46.1% (295/640) of CpG sites in HCC DNA and 5.4% (29/542) of CpG sites in matched non-HCC DNA showed “C only” in their antisense strand sequencing chromatograms (Fig. 2A). Although methylation of the antisense stand of the *APC* gene was not specific to HCC (specificity, 19%), the methylation density of the antisense strand in HCC tissues was significantly higher than that in normal liver tissue or in adjacent non-HCC tissues (*P*<0.0001) as analyzed by the Pearson χ^2^ test (Fig. 2B).

To confirm that methylation of the antisense strand of the *APC* gene increased with HCC, as suggested by BSP sequencing (Fig. 2B), we used a different approach, BSP cloning and sequencing, for the antisense strands from 4 normal livers, 4 HCC, and 4 matched adjacent non-HCC tissue samples. We sequenced 7 to 19 individual clones from each DNA sample. Because the BSP product for DNA sequencing was derived from an individual clone, the sequencing chromatogram showed either a “T only” or “C only” peak for each CpG site examined. This result differed from that obtained with the DNA isolated from liver tissue where a mix of “C” and “T” peaks was often observed (Fig. 1C). The representative sequencing results from each group are shown in Figure S1 (A–C), and the percentage of clones that were methylated at each CpG site (percent methylation) in each DNA sample and in each tissue group was calculated as summarized in Figure S1D, graphed in Figure S1E, and analyzed by the Pearson χ^2^ test. Consistent with the data from BSP sequencing (Fig. 2B), the density of methylation of the antisense strand in HCC tissues as determined by BSP cloning and sequencing was significantly higher than that in normal liver tissue and adjacent non-HCC tissue (*P*<0.0001).

### Methylation-specific PCR assays confirm antisense strand-biased methylation of the *APC* gene in normal liver; only methylation of the sense strand is HCC-specific

To confirm that existence of antisense strand-biased methylation of the *APC* gene in normal liver resulted in inconsistent HCC specificity, as suggested by BSP sequencing, and to evaluate the specificity of *mAPC* sense and *mAPC* antisense as potential biomarkers of HCC, we developed quantitative MSP assays for the sense strand (APC_MSP_S) and antisense strand (APC_MSP_AS) of the *APC* gene. The APC_MSP_S assay targets CpG sites 7–17 on the sense strand, whereas the APC_MSP_AS targets CpG sites 11–18 on the antisense strand. Primers and Taqman probe sequences for these two MSP assays are listed in Table S1. The sensitivity and linearity of these two MSP assays were determined using serial reconstituted samples, as described in Materials and Methods. Both assays exhibited linear amplification characteristics with the limit of detection of 6 copies per assay (Fig. S2).

Next, we determined the extent of methylation of normal and diseased liver DNA on both sense and antisense strands of the *APC* gene, using two quantitative MSP assays. Unlike BSP sequencing, MSP assays are easier and less expensive to perform. We included additional samples of hepatitis (n = 39), cirrhosis (n = 41), and HCC (n = 58) so that the performance of this potential biomarker could be evaluated with sufficient statistical power. This group of subjects included those studied by BSP sequencing; clinical information regarding tumor stage, grade, and serum level of AFP is summarized in Table 3. To perform MSP assays, as detailed in Materials and Methods, we first quantified each bisulfite-treated DNA sample using a BS-actin PCR assay. Next, approximately 150 copies of each DNA sample were subjected to MSP assays for the sense and antisense strands of the *APC* gene, respectively, using reconstituted standards. The amount of methylated DNA from each input sample was obtained by referencing the standard curve generated.

**Table 3:** Summary of clinicopathological characteristics of the tissues analyzed by the MSP assays.

Characteristic	Normal†(n = 6)	Hepatitis(n = 39)	Cirrhosis(n = 41)	HCC and adjacent non-HCC(n = 58)	*P* value
**Mean age ± SD** ◊	64±7.5	55±11.9	56±14.4	58.69±11.89	0.19§
**Male/female**	4/2	19/20	27/14	39/19	0.298§
**HBV/HCV/others**	0/0/0	12/31/5	6/22/13	30/15/13	-
**Stage** §§ **1/2/3/4/unknown**	-	-	-	28/21/3/1/5	-
**Grade 1/2/3/unknown**	-	-	-	11/33/9/5	-
**Mean size of tumor ± SD**	-	-	-	5.89±4.09 cms	-
**AFP levels (ng/ml)** **>20/≤20/unknown**	-	-	-	30/23/5	-

◊ SD, standard deviation.

§ Across all subjects (n = 144), age was analyzed by the Student *t* test and gender, by the Fisher exact test.

§§ HCC tumors were staged using TNM staging system.

† 4 of these 6 normal livers are normal liver tissues with concomitant cholangiocarcinoma.

### *APC* sense strand methylation as a DNA marker and its detection in AFP-negative HCC

**Figure 3:**
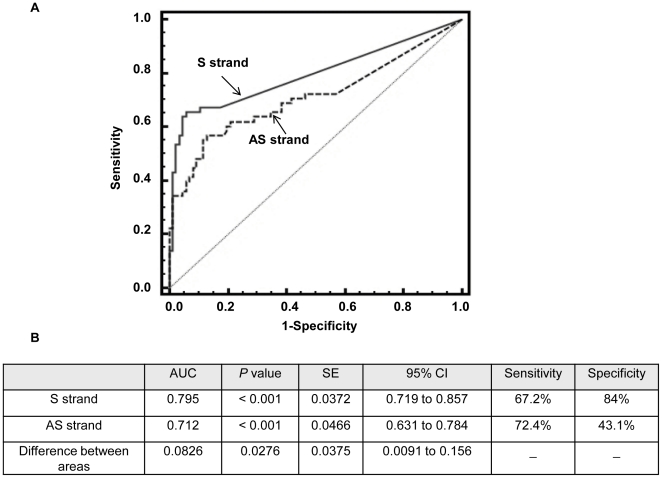
Receiver operating characteristic (ROC) curves of sense strand methylation (solid line, panel A) or antisense strand methylation (dashed line, panel A) of the *APC* gene as a marker to discriminate HCC (n = 58) from all non-HCC liver tissues including normal (n = 6), hepatitis (n = 39), cirrhosis (n = 41), and matched adjacent non-HCC (n = 58). The amount of methylated DNA was the average of two duplicate MSP assays as detailed in Materials and Methods. The area under the curve of each ROC curve and statistical analyses are shown in panel B.

As suggested previously, methylation of the *APC* gene is a potential biomarker for HCC detection, despite the fact that some studies showed little or no HCC specificity when the antisense strand of DNA was analyzed. To evaluate the performance of *mAPC* as a marker to distinguish HCC from non-HCC tissues including normal, hepatitis, cirrhosis, and non-HCC liver from HCC patients, we constructed receiver operating characteristic (ROC) curves for both sense and antisense *mAPC* using data generated by the quantitative MSP assays developed in this study (Fig. 3). The area under the curve (AUROC) was calculated as 0.795 for sense *mAPC* and 0.712 for antisense *mAPC*. Pairwise comparison of the two ROC curves yielded a *P* value of 0.0276, suggesting that there was no significant difference between sense and antisense *mAPC* as biomarkers in distinguishing between HCC and non-HCC liver tissues. However, when we defined a cutoff value as any detectable methylation per 150 copies input DNA by MSP assay, we observed a significant difference in the specificity of sense *vs.* antisense strand methylation, as shown in Figure 3B. Little or no specificity (43%) was observed when we analyzed the antisense strand, whereas very high specificity (84%) was obtained when the sense strand was analyzed. This result is consistent with previous reports listed in Table 1, in which end-point MSP was analyzed. The sensitivities of both sense and antisense methylation as biomarkers for HCC were comparable (67% *vs.* 72%).

**Figure 4:**
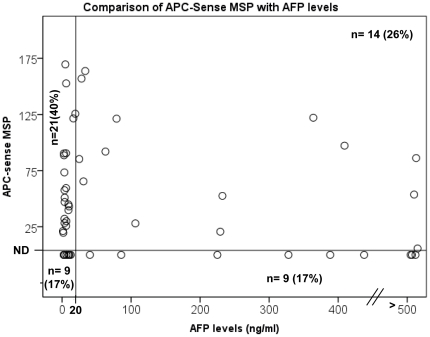
Scatter plot distribution of serum AFP levels (y-axis) and the amount of methylated sense strand *APC* DNA for 53 HCC samples. Each circle represents the value for an individual HCC case. A vertical reference line intersects at 20 ng/ml AFP value. A horizontal reference line intersects right above the MSP value of 0 as the reference for undetectable (ND), which is less than 10 copies per assay. The number of HCC cases and the percent of the total HCC in each of four areas are indicated.

Because sense strand *mAPC* exhibited significantly higher specificity as a biomarker for HCC when a cutoff value was applied and *mAPC* was detected in the blood of patients with HCC, suggesting it as a potential circulating DNA marker for HCC screening [36], [37], we compared sense *mAPC* with the current accepted biomarker for HCC screening, the serum AFP level. A scatter plot was generated by plotting the serum AFP level (ng/mL) on the x-axis and the sense *mAPC* level in HCC on the y-axis (Fig. 4). According to the American Association for the Study of Liver Diseases, positive AFP for HCC is defined as ≥20 ng/mL; in our study population, 43% of HCC tissues (n = 53 for which AFP levels were available) were positive for AFP. We found 66% (35/53) of HCC samples to be positive for sense *mAPC*. When we combined these two biomarkers, 83% of HCC samples were positive for at least one marker. Interestingly, 60% of *mAPC*-positive HCCs (21/35) were negative for AFP. Spearman's correlation was used to compare sense *mAPC* and AFP, suggesting that these two factors are not correlated or are independent of each other (*P* = 0.535).

### The relationship between the status of the APC sense strand methylation and clinicopathological variables

The methylation status of the sense strand of the *APC* gene as determined by the MSP assay was compared to various clinicopathological variables and the major etiologies of HCC as summarized in Table 4. The clinicopathological data for five subjects were not available, and these subjects were not included in the analysis. A comparison of stage 1 (n = 28) with stages 2 through 4 (n = 21, 1, and 5, for stages 2, 3, and 4, respectively) indicated that *mAPC* was significantly higher (*P* = 0.017) in stage 1 than in the later stages (stages 2–4). No significant difference was found between low (grade 1) and high (grade 2 and 3) tumor grades (*P* = 0.401), AFP-negative and AFP-positive tumors (*P* = 0.963), HBV-infected and non-HBV-infected HCC (*P* = 0.985), and/or HCV status (*P* = 0.445).

**Table 4:** Statistical analysis of the amount of sense *mAPC* in each subclinical group of HCC patients.

Comparison of HCC samples (n = 53)	*P* value
Stage§ (1 vs 2,3,4)	0.017*
Grade (1 vs 2,3)	0.401
HBV status	0.985
HCV status	0.445
AFP levels (<20, >20)	0.963

* P<0.05.

§ HCC tumors were staged using the TNM staging system.

### *APC* antisense strand-biased methylation is liver tissue-specific and does not occur in murine liver

**Figure 5:**
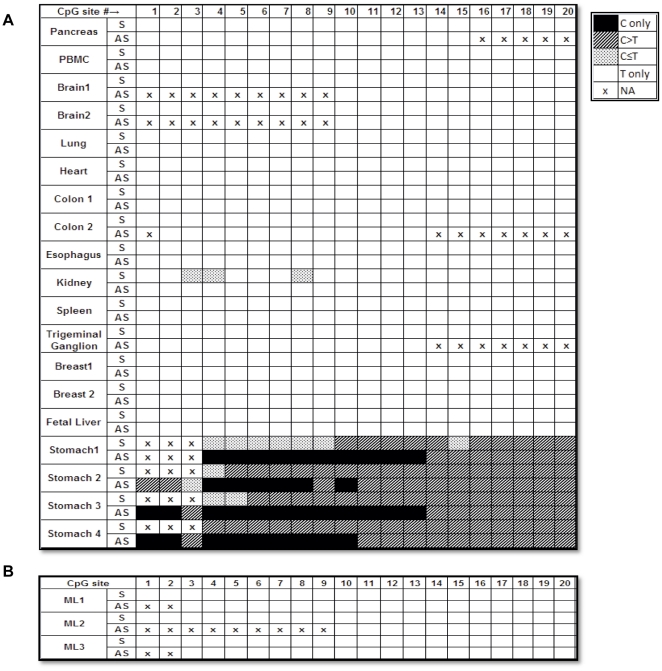
Methylation profiles for sense (S) and antisense (AS) strands of the promoter and the first exon regions of the *APC* gene in nonliver normal human tissues (A) and mouse normal liver (B). DNA sequencing data were obtained using BSP sequencing and analyzed as described in Figure 1.

To our knowledge, antisense strand-biased methylation has not been reported previously. Therefore, to determine whether the observed novel finding was tissue-specific, we analyzed DNA isolated from thirteen different normal tissue types: pancreas, peripheral blood mononuclear cells, brain, trigeminal ganglion, lung, heart, colon, esophagus, stomach, kidney, breast, spleen, and fetal liver. Because direct BSP sequencing indicates the net overall methylation status of DNA and avoids differential biases that can occur during bacterial cloning, we used BSP sequencing to compare and analyze other tissue samples using the reference index that we established (Fig. 1). As shown in Figure 5A, we did not detect any antisense strand-biased methylation in any of the nonliver tissues or fetal liver tissues studied. Methylation of the promoter 1A of the *APC* gene occurred in normal gastric DNA in a monoallelic and age-dependent but not in an antisense strand-biased manner [38], [39], [40]. By studying stomach tissue DNA from four individuals of different ages, 27, 29, 50, and 58 years, we confirmed that the *APC* gene is methylated in normal gastric tissue DNA but in an antisense strand-biased manner.

Mice are frequently used as animal models for studying human diseases, including cancers. Demethylating agents have been tested in mice as potential anticancer drugs [41]. Thus, it was of interest to determine whether antisense strand-biased methylation of the *APC* gene also occurred in mouse liver. Liver tissue DNA from three individual BALB/c female mice was subjected to BSP direct sequencing. No ^m^CpG was detected in the three mice livers studied (Fig. 5B).

## Discussion

Four major findings are discussed in this study. First, we discovered an antisense strand-biased methylation pattern in the CpG island of the promoter and first exon regions of the tumor suppressor gene *APC* in normal liver. This finding supports our hypothesis that the previously reported variable HCC specificity of *mAPC*, as summarized in Table 1, may have been due to differences in methylation status between sense and antisense strands of *APC* in normal and diseased liver tissues. Second, although antisense strand methylation occurs in normal liver, methylation related to HCC carcinogenesis occurs on both strands. The density of both antisense and sense strand methylation increased significantly in HCC (*P*<0.0001) compared to non-HCC tissues; thus, the ROC curves of sense *mAPC* and antisense *mAPC* are not statistically different. However, given the background level of antisense methylation of the *APC* gene that exists in normal liver, methylation of the sense strand should be used to distinguish HCC from non-HCC for a higher specificity, particularly when end-point MSP is performed.

Third, antisense strand-biased methylation of the *APC* gene was not observed in any of the 13 nonliver normal human tissues studied, suggesting a liver tissue-specific epigenetic pattern. It has been reported that CpG methylation may differ among different tissues and can be associated with tissue-specific expression [42], [43]. The liver-specific antisense strand-biased methylation of the *APC* gene shown in this study serves to further suggest that DNA methylation in a tissue-specific manner should be taken into consideration when studying disease-related epigenetic events.

Lastly, when comparing the sense *mAPC* to the currently accepted HCC marker, serum AFP level, we found that these two markers are independent, as shown by Spearman's correlation test. The sense *mAPC* marker identified an additional 40% of the HCC cases in our study population that would otherwise have been missed by serum AFP level alone. Currently, no biochemical marker is available for detecting AFP-negative HCC, which constitutes approximately 50% of HCC cases in the general population [44]. Although the *mAPC* detected in this study was found in HCC tissue, *mAPC* DNA has been detected previously in the circulation of patients with HCC [36], [45]. It is encouraging that *mAPC* could be a potential biomarker complementary to AFP in identifying AFP-negative liver tumors and that it occurs frequently in early stage (stage 1) HCC (Table 4), which is important for the early detection of HCC. Interestingly, we did not find a significantly higher incidence of *mAPC* in HBV-infected HCC than non-HBV-infected HCC in this study, nor in HCV-infected HCC, although it has been suggested that HBV or HCV infection increases the aberrant methylation of tumor suppressor genes in HCC, including the *APC* gene [32], [46]. This discrepancy could be due to the differences in clinicopathological characteristics of the HCC tumors used in different studies.

The antisense strand-biased methylation pattern of *APC* appeared in most of the non-HCC liver tissues analyzed except for cirrhosis, in which only two of six cirrhotic tissues were found to exhibit antisense strand-biased methylation by BSP sequencing. Cirrhotic tissue is a mixture of regenerated hepatic tissue and scar tissue. It is possible that the tissue used for DNA isolation was predominantly fibrotic tissue and that relatively insensitive BSP direct sequencing was therefore unable to detect strand-biased methylation in the small hepatic component. One of the six cirrhotic tissue samples exhibited symmetrical methylation similar to that of the HCC samples. This result was not surprising, since other researchers have also reported *APC* promoter methylation in cirrhotic tissues [4], [5]. Because we did not detect any strand-biased ^m^CpG in DNA isolated from any of the other 13 tissues tested, including fetal liver, we concluded that antisense strand-biased methylation of the *APC* gene occurs uniquely in adult liver. We did not find any evidence of *APC* antisense strand-biased methylation in murine liver tissue. This result may have implications regarding the extrapolation of results in humans to experiments in mouse models, such as the testing of potential anticancer drugs with demethylating properties.

Cancer is a disease of the genome and epigenome; thus, the detection of genetic and epigenetic changes underlying the development of HCC should aid in the unambiguous detection of tumors. Interestingly, one of the potential HCC epigenetic DNA markers we examined, *mAPC*, exhibits liver-specific methylation patterns, suggesting that, in the search for epigenetic DNA markers for detection of HCC, the methylation status of normal liver in both the sense and antisense strands should be taken into consideration when developing a sensitive and specific assay for the detection of HCC.

## Materials and Methods

### Subjects

The archived DNA isolated from 66 HCC tissue samples and adjacent non-HCC liver samples used in this study was obtained with written informed consent from patients who underwent radical resection at Zhong Shan Hospital, Shanghai, China (n = 8) and the National Cheng-Kung University Medical Center, Tainan, Taiwan (n = 58) in accordance with the guidelines of the respective institutional review boards. The institutional review boards of Drexel University College of Medicine, National Cheng-Kung University Medical Center, and Zhong Shan Hospital specifically approved this study.

The archived DNA isolated from 4 normal liver (N3–N6), 4 hepatitis-infected liver (H1–H4), 6 cirrhotic liver (C1–C6), esophageal, and colonic tissues was obtained from the Johns Hopkins University School of Medicine in accordance with Institutional Review Board protocols. Additional archived DNA samples (35 hepatitis and 35 cirrhosis) were obtained from the Buddhist Tzu Chi Medical Center, Hualien, Taiwan, in accordance with Institutional Review Board protocols. Archived DNA from normal liver sample #N1 and from heart and lung tissue samples obtained from the National Disease Research Interchange, Philadelphia was donated by Immunotope, Inc. (Doylestown, PA).

Thirty-two of the matched HCC tissue samples were used for BSP and sequencing, and 58 of the matched HCC tissue samples were analyzed by MSP. The profiles for this group are shown in Tables 2 and 3. Normal peripheral blood mononuclear cell DNA was obtained as a gift from the laboratory of Dr. Pooja Jain (Drexel University College of Medicine, Philadelphia, PA). Normal liver N2 tissue DNA was purchased from Capital Biosciences (Rockville, MD), and stomach 1–4, pancreas, kidney, spleen, breast, brain, trigeminal ganglion, and fetal liver DNAs were purchased from the BioChain Institute (Hayward, CA). Individual subject information is listed in Table S3. Mouse livers from Balb/c female mice were obtained from Charles River Laboratories (Wilmington, MA).

### DNA isolation and bisulfite treatment

DNA was isolated using Qiagen DNeasy Blood and Tissue kits (Qiagen, Valencia, CA) according to the manufacturer's instructions. DNA concentration was measured using a Nanodrop 1000 spectrophotometer (Thermo Fisher Scientific, Inc., Wilmington, DE) at 260 nm absorbance. Bisulfite treatment was performed using Qiagen EpiTect Bisulfite conversion kits (Qiagen) following the guidelines of the manufacturer.

### Bisulfite-specific PCR sequencing

BSP primers were designed using Methyl Primer Express software (Applied Biosystems, Life Technologies Corp, Carlsbad, CA) to amplify the promoter regions of the *APC* (APC_BSP_S, APC_BSP_AS1,and APC_BSP_AS) for both the sense and antisense strands; primer sequences are described in Table S1. PCR was performed in an Eppendorf Mastercycler thermocycler for 40 cycles with hot-start Taq polymerase (Qiagen). The PCR program started with activation of the polymerase at 95°C for 15 min, followed by denaturation at 95°C for 30 s, annealing at the respective annealing temperature (Table S1) for 30 s, and extension at 72°C for 30 s, then followed by a final 4-min extension at 72°C and cooling at 4°C for all primer sets. The reaction was assembled in a final volume of 20 µl containing 0.5 U HotStart Taq (Qiagen), 1× PCR buffer, 200 µM of dNTPs, 0.5 µM each of primer, and bisulfite-treated DNA templates. PCR products were run on 1% agarose gel with 1× TAE buffer. The PCR product of the correct size was excised, purified with a Qiagen Gel Purification kit (Qiagen), and sent with the appropriate primer for sequencing to the NAPCore facility at the Children's Hospital of Philadelphia, Philadelphia, PA. Sequencing results were analyzed using ClustalW software (available at http://www.ch.embnet.org/), the Chromas 2.3 software (Technelysium, Tewantin, Queensland, Australia), and Finch TV version 1.4.0 (GeospizaInc, Seattle, WA).

### Preparation of reconstituted standards of methylated and unmethylated DNA for BSP sequencing and MSP assays

To determine the sensitivity of BSP sequencing and MSP assays to detect methylated DNA and estimate the relative amount of methylated DNA in a given sample, we prepared a reconstituted sample set (*i.e.*, a known amount of methylated DNA in a background of unmethylated DNA). Bisulfite-converted human universal methylated DNA control (Zymo Research, Seattle, WA) was used as the methylated DNA standard. Bisulfite-treated DNA from normal human peripheral blood mononuclear cells that was confirmed by sequencing to be unmethylated in the *APC* region of interest was used as a source of unmethylated DNA. To quantify bisulfite-converted DNA for both methylated and unmethylated control DNA, we developed a real-time PCR assay, BS-actin, targeting the BS-converted actin gene sequences. The primers of the BS-actin PCR, listed in Table S1, were designed within regions lacking CpG sites, so that CpG methylation status would not affect primer binding. This assay was tested for linearity and sensitivity using 10-fold dilutions of bisulfite-converted human universal methylated control DNA (Zymo Research). A conversion factor of 6 pg of DNA per cell was used to calculate the amount of DNA. On the basis of quantification by BS-actin PCR, reconstituted sample sets were prepared in the following ratios: (1) 0% methylated DNA, 100% unmethylated DNA; (2) 10% methylated DNA, 90% unmethylated DNA; (3) 25% methylated DNA, 75% unmethylated DNA; (4) 50% methylated DNA, 50% unmethylated DNA; and (5) 100% methylated DNA.

### BSP cloning and sequencing

BSP cloning and sequencing were performed for four normal liver samples (N2, N3, N5, N6) and four sets of HCC plus matched adjacent non-HCC bisulfite-treated DNA samples (HCC 2–4, HCC6, non-HCC 2–4, non-HCC 6). BSP products obtained from the APC_BSP_AS1 and the APC_BSP_AS2 reactions were gel-purified using a Qiagen Gel Purification kit (Qiagen) followed by polishing, ligation, and transformation performed according to the protocols of the PCR-script Amp Cloning kit (Stratagene, Santa Clara, CA). The white colonies were screened for the insert using T3 and T7 primers. The PCR product obtained from each positive clone thus isolated was then gel-purified using a Qiagen Gel Purification kit (Qiagen) and sent for sequencing to the NAPCore facility at the Children's Hospital of Philadelphia. Sequencing results were analyzed using ClustalW software (available at http://www.ch.embnet.org/) and Chromas 2.3 software (Technelysium). Seven to nineteen clones were sequenced for each sample.

### Methylation-specific PCR assay

Two quantitative methylation-specific PCR assays for the sense strand (APC_MSP_S) and the antisense strand (APC_MSP_AS) of the *APC* gene were developed. The primer pairs and TaqMan probes of these two assays are shown in Table S1. For the APC_MSP_S assay, a 10-µl reaction was assembled using FastStart TaqMan Probe Master mix (Roche Applied Science, Mannheim, Germany). This reaction contained 1× FastStart TaqMan Probe Master mix, 1.0 µM primers, 2.5 mM MgCl_2_, and the DNA template. Using a Roche LightCycler 480 Real-Time PCR system, PCR reactions were performed under the following conditions: 95°C 10 min (95°C×10 s, 65°C×30 s, 72°C×10 s)×50 cycles, 40°C×30 s. For the APC_MSP_AS assay, a 10-µl reaction was assembled using LightCycler Taqman Master mix (Roche Applied Science, Mannheim, Germany). This reaction contained 1× Taqman Master Mix, 1.0 µM primers, 2.5 mM MgCl_2_, and the DNA template. Using a Roche LighCycler 2.0 Real-Time PCR system, PCR reactions were performed under the following conditions: 95°C 10 min (95°C×10 s, 56°C×15 s, 72°C×10 s)×50 cycles, 40°C×30 s. Approximately 150 copies of DNA, as estimated by the BS-actin PCR assay, were used in each MSP reaction in duplicate. The average of two assays was used for the analysis.

### Statistical analysis

Methylation density for BSP sequencing and BSP cloning and sequencing was evaluated statistically using a two-sided Pearson χ^2^ test to compare HCC with adjacent non-HCC liver and HCC with normal liver from control patients. Contingency tables were constructed for each comparison group (e.g., HCC *vs.* adjacent non-HCC liver) containing the total number of sites in each of the four methylation density groups (C only, C>T, C<T, or T only). For BSP sequencing, analysis was performed in two ways: (i) including data for all available CpG sites; and (ii) ignoring CpG sites that had data unavailable for any of the samples. For HCC *vs.* normal liver, sites #3 to #6 and #10 to #17 were used; for HCC *vs.* adjacent non-HCC liver, sites #2 to #17 were used. For BSP cloning and sequencing methylation density analysis, the total number of methylated CpG sites for each tissue group (HCC *vs.* normal liver and HCC *vs.* adjacent non-HCC liver) was compared using the Pearson χ^2^ test.

To test whether age and gender were evenly distributed across both HCC and non-HCC groups, the Student *t* test was performed for age and Fisher's exact test was performed for gender. To study the distribution of *APC* sense-MSP values in HCC tissues across the categories of stage, grade, HBV status, HCV status, and AFP groups (<20 or >20 ng/ml), a Kruskal-Wallis test was performed. Stages 2, 3, and 4 were combined into one group, and Grades 2 and 3 were combined into one group because the numbers of samples in stage 3 (n = 3), stage 4 (n = 1), and grade 3 (n = 9) were low. To study the correlation between AFP levels and *APC* sense MSP values, Spearman's correlation test was used. Receiver-operating characteristic (ROC) curves, areas under the ROC curves, and comparisons between ROCs were generated using MedCalc for Windows, version 11.5.0.0 (MedCalc Software, Mariakerke, Belgium). The scatter plot distribution of serum AFP levels (y-axis) and of the amount of methylated sense strand *APC* DNA distribution was constructed using the PASW software (IBM, New York).

## Supporting Information

Figure S1 **Methylation density of each CpG site obtained by DNA sequencing of each BSP clone.** An example of DNA sequencing results obtained from 14 BSP clones isolated from cloning the BSP product derived from normal liver sample 2 (A), 19 BSP clones from HCC sample 6 (B), and 17 BSP clones from adjacent non-HCC sample 6 (C). The percent of methylation for each CpG site was calculated using the number of ^m^CpGs detected per total number of clones analyzed, as listed at the bottom of the figure. D, summary of the percent of methylation for each CpG site from DNA isolated from 4 normal livers, 4 HCC samples, and the matched adjacent non-HCC tissue. E, histogram showing the percentage of each CpG site methylation for all CpG sites as tabulated in D (***P<0.0001 for HCC *vs.* normal liver and ***P<0.0001 for HCC *vs.* adjacent non-HCC). E, The methylation density of the antisense strand of the APC promoter and first exon regions increase with HCC as determined by BSP cloning and sequencing. The methylation density (percent of methylated CpG sites) of each CpG site of each indicated DNA sample as summarized in panel D. The data are plotted per CpG site on the y-axis of each DNA sample and analyzed by the Pearson χ^2^ test. *** indicates *P*<0.0001.
(TIF)

Figure S2 **Amplification and standard curves of the sense (A) and antisense (B) MSP assays.** Serial 1∶10 dilutions of human methylated bisulfite-converted genomic DNA were amplified by the *APC* sense and antisense MSP assays as detailed in Materials and Methods. The curves generated by different amounts of input DNA (copies) per reaction are indicated.
(TIF)

Table S1 Primer and probe sequences used for bisulfite DNA sequencing and methylation-specific PCR for both sense and antisense DNA strands (Genbank accession number: APC: NG_0084811).
(DOCX)

Table S2 Percent of mCpG detected in the antisense strand of the promoter and the first exon regions of the APC gene in each pathological group of liver tissue*.
(DOCX)

Table S3 **Subject information for nonliver tissues.**
(DOCX)
