# Supplementary figures and images for: Methylation of the CpG Sites Only on the Sense Strand of the APC Gene Is Specific for Hepatocellular Carcinoma
Source: PLoS One. 2011 Nov 2;6(11):e26799. doi: 10.1371/journal.pone.0026799 (PMC3206845; doi:10.1371/journal.pone.0026799)

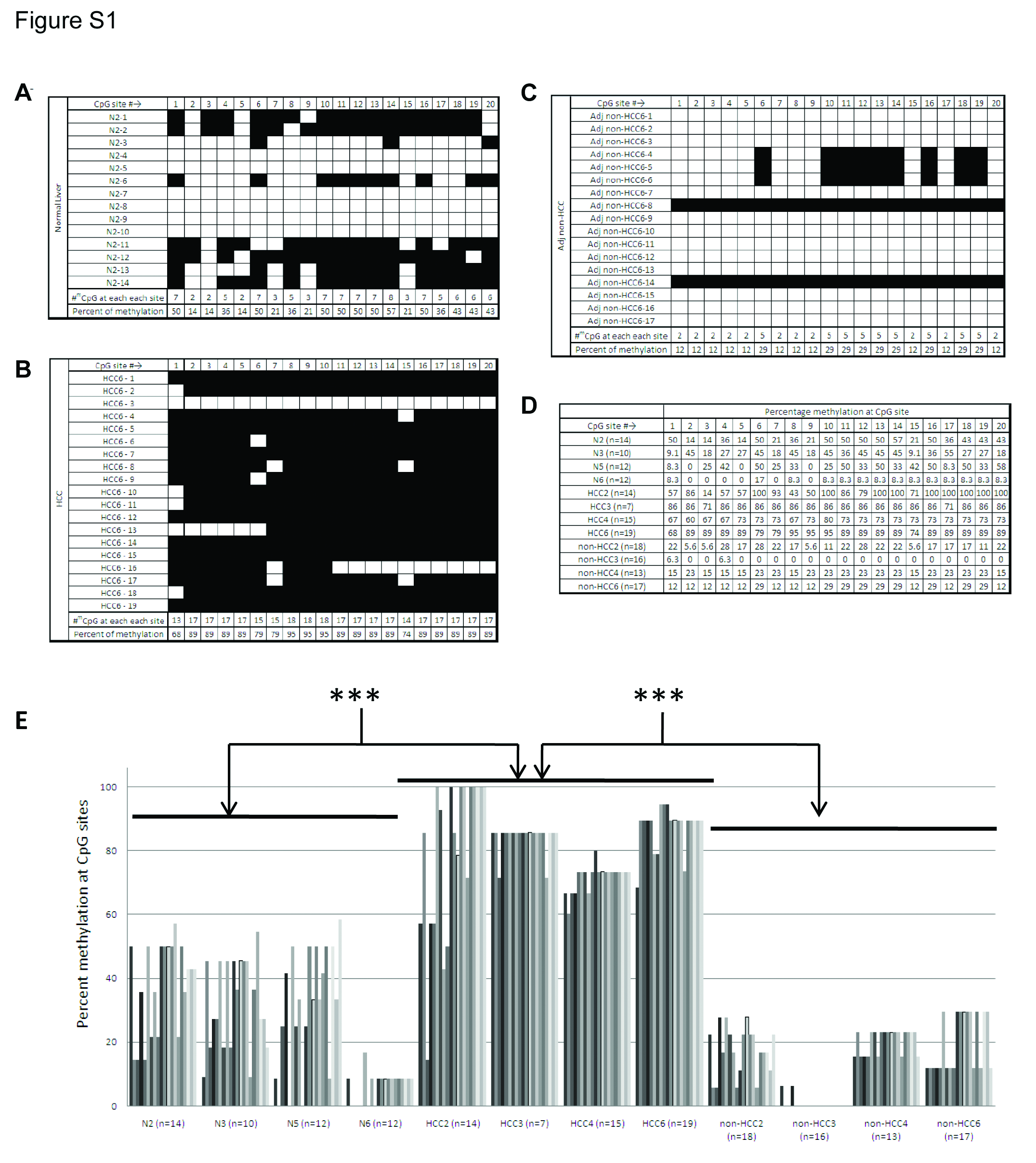

Supplement: Figure S1 — Methylation density of each CpG site obtained by DNA sequencing of each BSP clone. An example of DNA sequencing results obtained from 14 BSP clones isolated from cloning the BSP product derived from normal liver sample 2 (A), 19 BSP clones from HCC sample 6 (B), and 17 BSP clones from adjacent non-HCC sample 6 (C). The percent of methylation for each CpG site was calculated using the number of mCpGs detected per total number of clones analyzed, as listed at the bottom of the figure. D, summary of the percent of methylation for each CpG site from DNA isolated from 4 normal livers, 4 HCC samples, and the matched adjacent non-HCC tissue. E, histogram showing the percentage of each CpG site methylation for all CpG sites as tabulated in D (***P<0.0001 for HCC vs. normal liver and ***P<0.0001 for HCC vs. adjacent non-HCC). E, The methylation density of the antisense strand of the APC promoter and first exon regions increase with HCC as determined by BSP cloning and sequencing. The methylation density (percent of methylated CpG sites) of each CpG site of each indicated DNA sample as summarized in panel D. The data are plotted per CpG site on the y-axis of each DNA sample and analyzed by the Pearson χ2 test. *** indicates P<0.0001. (TIF) [file pone.0026799.s001.tif]

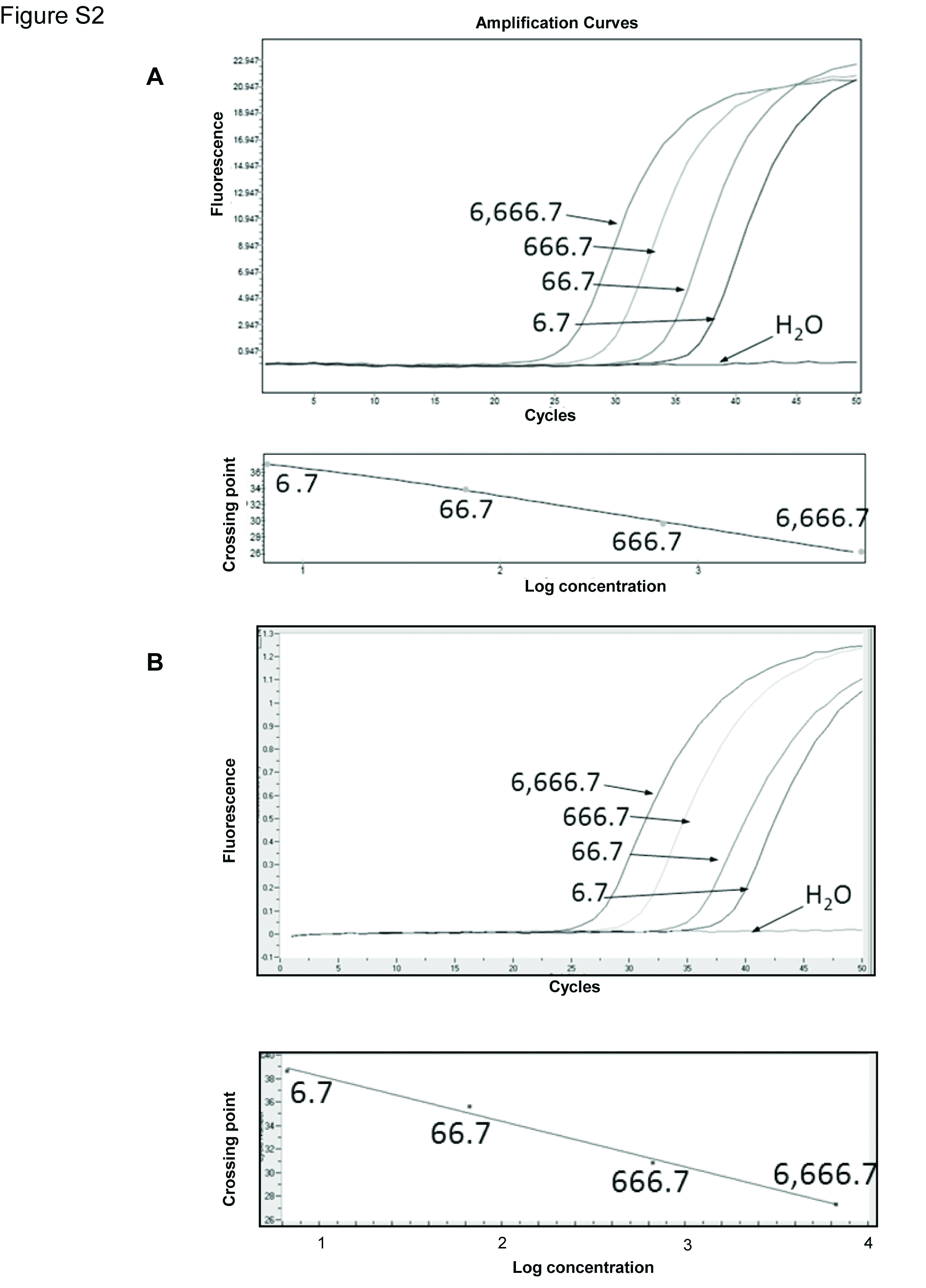

Supplement: Figure S2 — Amplification and standard curves of the sense (A) and antisense (B) MSP assays. Serial 1∶10 dilutions of human methylated bisulfite-converted genomic DNA were amplified by the APC sense and antisense MSP assays as detailed in Materials and Methods. The curves generated by different amounts of input DNA (copies) per reaction are indicated. (TIF) [file pone.0026799.s002.tif]
