# Supplementary material for: Methylation of the CpG Sites Only on the Sense Strand of the APC Gene Is Specific for Hepatocellular Carcinoma
Source: PLoS One. 2011 Nov 2;6(11):e26799. doi: 10.1371/journal.pone.0026799 (PMC3206845; doi:10.1371/journal.pone.0026799)
Supplement: Table S1 — Primer and probe sequences used for bisulfite DNA sequencing and methylation-specific PCR for both sense and antisense DNA strands (Genbank accession number: APC: NG_0084811). (DOCX) [file pone.0026799.s003.docx]

Table S1

| **Gene/PCR** | **Strand** | **Primers and probe sequence** | **Location** | **Annealing temperature** |
| --- | --- | --- | --- | --- |
| APC/BSP | S | F: atttttttgtttgttggggatt | 35105-35585 | 56 |
|  |  | R: ggaaatttatttttagtgttgtag |  |  |
|  | AS-1 | F1: acaaatcatcactctaacaactcaat | 34809-35346 | 55 |
|  |  | R1: aagtttggttatggtggtttta |  |  |
|  | AS-2 | F2: atcaactaccatcaacttccttac | 35101-35383 | 56 |
|  |  | R2: ttttagtgatattttggygggttg |  |  |
| APC/MSP | S | F: tattgcggagtgcgggtc | 35,205-35,222 | 65 |
|  |  | R: tcgacgaactcccgacga |  |  |
|  |  | Probe: 5′-FAM-aaaacgccctaatccgcatccaacg-BHQ1-3′ |  |  |
|  | AS | F: tgcgtttatatttagttaatcggc | 35,246-35,321 | 56 |
|  |  | R: gaaatacgaatcgaaaaacgaa |  |  |
|  |  | Probe:5’-FAM- acgctccccattcccgtcga-BHQ1-3’ |  |  |

*APC*, adenomatous polyposis coli; AS, antisense; BSP, bisulfite-specific PCR; MSP, methylation-specific PCR; PCR, polymerase chain reaction; S, sense **0% 100% 100%**
