# Supplementary material for: Methylation of the CpG Sites Only on the Sense Strand of the APC Gene Is Specific for Hepatocellular Carcinoma
Source: PLoS One. 2011 Nov 2;6(11):e26799. doi: 10.1371/journal.pone.0026799 (PMC3206845; doi:10.1371/journal.pone.0026799)
Supplement: Table S2 — Percent of mCpG detected in the antisense strand of the promoter and the first exon regions of the APC gene in each pathological group of liver tissue*. (DOCX) [file pone.0026799.s004.docx]

Table S2

| Density | Symbol | HCC | Adjacent non-HCC | Cirrhosis | Hepatitis | Normal liver |
| --- | --- | --- | --- | --- | --- | --- |
|  |  |  |  |  |  |  |
| C only |  | 295 (46.1%) | 29 (5.4%) | 0 (0%) | 0 (0%) | 0 (0%) |
| C > T |  | 239 (37.4%) | 228 (42.0%) | 27 (22.7%) | 48 (60%) | 47 (48.9%) |
| C ≤ T |  | 59 (9.2%) | 161 (29.7%) | 20 (16.8%) | 24 (30%) | 28 (29.1%) |
| T only |  | 47 (7.3%) | 124 (22.9%) | 72 (60.5%) | 8 (10%) | 21(21.8%) |
| Total** |  | 640 | 542 | 119 | 80 | 96 |

*****The symbol and analysis of the methylation status of the CpG sites are described in Figure 1B. The percent of ^m^CpG detected is calculated from the number of each category per pathological group. For example, 295 CpG sites were detected as “C only, filled box” among the total 640 sites analyzed in the HCC group (Fig. 2); thus, the percent of “C only” is 46.2% in the HCC group.

**Total number of CpG sites analyzed.

*APC*, adenomatous polyposis coli; HCC, hepatocellular carcinoma; ^m^CpG, methylated cytosine in a CpG dinucleotide
