# Supplementary material for: Methylation of the CpG Sites Only on the Sense Strand of the APC Gene Is Specific for Hepatocellular Carcinoma
Source: PLoS One. 2011 Nov 2;6(11):e26799. doi: 10.1371/journal.pone.0026799 (PMC3206845; doi:10.1371/journal.pone.0026799)
Supplement: Table S3 — Subject information for nonliver tissues. (DOCX) [file pone.0026799.s005.docx]

Table S3

| Subject | Gender | Age, years | Pathological profile | HBV-infected | HCV-  infected |
| --- | --- | --- | --- | --- | --- |
| Pancreas | M | 27 | Normal | NA | NA |
| Kidney | F | 63 | Normal | NA | NA |
| Spleen | F | 83 | Normal | NA | NA |
| Stomach 1 | M | 27 | Normal | NA | NA |
| Stomach 2 | M | 50 | Normal | NA | NA |
| Stomach 3 | M | 58 | Normal | NA | NA |
| Stomach 4 | M | 29 | Normal | NA | NA |
| Brain 1 | M | 78 | Normal | NA | NA |
| Brain 2 | M | 75 | Normal | NA | NA |
| Lung | F | 50 | Normal | NA | NA |
| Heart | M | 67 | Normal | NA | NA |
| Colon 1 | F | 85 | Normal | NA | NA |
| Colon 2 | F | 77 | Normal | NA | NA |
| Esophagus | M | 73 | Normal | NA | NA |
| Trigeminal ganglion | M | 75 | Normal | NA | NA |
| Breast 1 | F | 78 | Normal | NA | NA |
| Breast 2 | F | 21 | Normal | NA | NA |
| Fetal liver | M | 29 weeks | Normal | NA | NA |

F, female; HCC, hepatocellular carcinoma; M, male; NA, not applicable
